# Predictive value of temporal muscle thickness in patients with chronic subdural hematoma

**DOI:** 10.3389/fneur.2025.1704557

**Published:** 2026-01-07

**Authors:** Jingzhe Yuan, Haoqi Zeng, Qingnan Wu, Weiming Liu, Yunwei Ou

**Affiliations:** 1 Department of Neurosurgery, Beijing Tiantan Hospital, Capital Medical University, Beijing, China; 2 Key Laboratory of Carcinogenesis and Translational Research (Ministry of Education/Beijing), Laboratory of Molecular Oncology, Peking University Cancer Hospital & Institute, Beijing, China; 3 The Institute of Artificial Intelligence, Hefei Comprehensive National Science Center, Hefei, Anhui, China

**Keywords:** chronic subdural hematoma, temporal muscle thickness, sarcopenia, prognostic marker, functional outcomes

## Abstract

**Background:**

Chronic subdural hematoma (CSDH) is a common neurosurgical condition that predominantly affects elderly individuals and is characterized by the accumulation of blood between the dura mater and the brain surface. Temporal muscle thickness (TMT) has emerged as a potential prognostic marker for various neurological diseases, including CSDH. This study aimed to evaluate the prognostic value of TMT in patients with CSDH.

**Methods:**

This retrospective study assessed TMT in patients with CSDH using cranial CT scans. We examined the correlation between TMT and CSDH-related clinical characteristics and evaluated the prognostic relevance of TMT.

**Results:**

A total of 844 participants were included in the study. TMT was significantly lower in patients with CSDH than in the control group (*p* < 0.001). The difference in TMT was strongly associated with the side of CSDH occurrence (*p* < 0.001), with CSDH more likely to develop on the side with lower TMT (OR = 0.219, 95% CI: 0.179–0.269). Lower average TMT was significantly associated with a higher likelihood of poor functional outcomes (OR = 0.560, 95% CI: 0.391–0.804, *p* = 0.002). Additionally, our analysis showed significant associations between TMT and age (*p* < 0.001), alcohol consumption (*p* = 0.009), cardiac disease (*p* = 0.006), brain infarction (*p* < 0.001), headache (*p* < 0.001), limb weakness (*p* < 0.001), and postoperative disturbance of consciousness (*p* = 0.029).

**Conclusion:**

The occurrence of CSDH is significantly correlated with the side of lower TMT. TMT may serve as an important prognostic marker in patients with CSDH, particularly for predicting functional outcomes.

## Introduction

Chronic subdural hematoma (CSDH) is a common neurosurgical condition characterized by the gradual accumulation of blood between the dura mater and the brain surface, often following minor head trauma. This hematoma compresses the underlying brain tissue, leading to various neurological symptoms (1, 2). In recent years, middle meningeal artery embolization (MMAE) has emerged as a promising minimally invasive technique for treating CSDH (3–6). The choice of treatment strategy depends on several factors, including the size and location of the hematoma, the patient’s symptoms, overall health, and the effectiveness of previous treatments (7). Typically, a multidisciplinary team collaborates to develop an individualized treatment plan for each patient (8). CSDH predominantly affects elderly individuals (9), presenting significant clinical challenges due to its high recurrence rate and associated morbidity. The reliability of currently identified prognostic factors requires further validation (10, 11). Therefore, identifying reliable prognostic indicators is essential for optimizing treatment strategies and improving patient outcomes.

Sarcopenia is defined as a progressive and generalized skeletal muscle disorder associated with an increased risk of adverse outcomes (12–15). In recent years, this concept has been incorporated into research on various cancers and other diseases related to sarcopenia (16–20). Temporal muscle thickness (TMT), as a surrogate marker of skeletal muscle function and sarcopenia risk, has received increasing attention and can be quantified using computed tomography (CT) scans. Recent studies have suggested that TMT may serve as a novel indicator for the clinical evaluation of physical condition and prognosis in patients with neurological diseases, including Parkinson’s disease, stroke, glioblastoma, lymphoma, non–small cell lung cancer with brain metastases, and melanoma with brain metastases (16, 21–26). These findings indicate that assessing the skeletal muscles of the head and neck, including TMT obtained from routine cranial CT scans in patients with CSDH, may serve as a novel prognostic approach that objectively reflects patients’ physical condition and prognosis.

This study aimed to retrospectively evaluate TMT in patients with CSDH using routine cranial CT. First, we assessed the correlation between TMT and CSDH-related clinical characteristics. Second, we evaluated the prognostic relevance and potential value of TMT as a surrogate marker.

## Materials and methods

### Patients and data collection

All patients admitted to the neurosurgical department of our institution between August 2011 and September 2017 with a diagnosis of CSDH were included in the analysis. Only unilateral CSDH patients were included to ensure population consistency, simplify the analysis, and avoid potential differences in pathophysiology between unilateral and bilateral CSDH. The inclusion criterion was a diagnosis of chronic subdural hematoma confirmed by CT scan. Patient characteristics and medical data were collected from the institutional electronic database. For this retrospective analysis, the Ethical Review Committee of Beijing Tiantan Hospital, Capital Medical University, approved the study design and protocol (ethics no. KY 2022-067-01) and waived the requirement for informed patient consent. The medical record parameters extracted included age, sex, Glasgow Coma Scale (GCS) score, anticoagulation status, preexisting conditions, symptoms, radiological parameters such as hematoma diameter and midline shift, clinical course, and status at discharge and 3 months after surgery.

### Image analysis

Preoperative cranial CT scans were analyzed using Neusoft PACS/RIS.^1^ Image analysis was performed by two neurosurgeons who were blinded to all clinical patient data. TMT was measured bilaterally for each patient on axial images at a standardized anatomical level, using the Sylvian fissure as the anterior–posterior landmark and the orbital roof as the cranio-caudal landmark. The left and right TMT values were measured separately; the average of the two was calculated to obtain the mean TMT, and the absolute difference between sides was defined as the TMT difference.

### Statistical analysis

Data analysis was performed using IBM SPSS Statistics version 29.0.1.0 (IBM Corp., Armonk, NY, United States). Descriptive statistics were used to summarize patient characteristics. For continuous parameters, the Mann–Whitney U test was used. Bivariable and multivariable logistic regression models were used to assess associations with TMT. Variables with a *p*-value ≤ 0.05 in the bivariable analysis were included in the multivariable logistic regression model. A p-value < 0.05 was considered statistically significant. To assess the effects of the variables, odds ratios (ORs) with 95% confidence intervals (CIs) were calculated. Results with *p* ≤ 0.05 were considered statistically relevant.

## Results

### Result 1 participant characteristics

A total of 844 participants were included in the study. The median age of the participants was 65 years (IQR: 54.5–75), with 701 (83.1%) males and 143 (16.9%) females. Regarding the side of CSDH occurrence, 479 patients (56.8%) had left-sided involvement and 365 patients (43.2%) had right-sided involvement. Among preexisting conditions, 553 patients (65.5%) had a history of trauma, 215 (25.5%) were smokers, 153 (18.1%) consumed alcohol, 300 (35.5%) had hypertension, 174 (20.6%) had diabetes, 26 (3.1%) had cardiac disease, 100 (11.8%) had a history of brain infarction, and 100 (11.8%) were receiving anticoagulation therapy. Regarding symptoms, 485 participants (57.5%) reported headaches, 229 (27.1%) experienced dizziness, 465 (55.1%) had limb weakness, 100 (11.8%) had dysphasia, and 38 (4.5%) presented with disturbances of consciousness. Outcomes measured using the modified Rankin Scale (mRS) showed that 828 participants (98.1%) had an mRS score of 0–3, indicating good recovery, whereas 16 (1.9%) had a score of 4–6, indicating severe disability or death (Table 1).

**Table 1:** Patient demographics and clinical characteristics.

Patient characteristic	(*n* = 844)
Age, median (IQR)	65 (54.5–75)
Sex	
Male, *n* (%)	701 (83.1)
Female, *n* (%)	143 (16.9)
Side of CSDH	
Left, *n* (%)	479 (56.8)
Right, *n* (%)	365 (43.2)
Preexisting conditions	
Trauma, *n* (%)	553 (65.5)
Smoking, *n* (%)	215 (25.5)
Drinking, *n* (%)	153 (18.1)
Hypertension, *n* (%)	300 (35.5)
Diabetes, *n* (%)	174 (20.6)
Cardiac disease, *n* (%)	26 (3.1)
Brain infarction, *n* (%)	100 (11.8)
Anticoagulation, *n* (%)	100 (11.8)
Symptoms	
Headache, *n* (%)	485 (57.5)
Dizziness, *n* (%)	229 (27.1)
Limb weakness, *n* (%)	465 (55.1)
Dysphasia, *n* (%)	100 (11.8)
Disturbance of consciousness, *n* (%)	38 (4.5)
Recurrence, *n* (%)	67 (7.9)
Outcomes	
mRS0-3, *n* (%)	828 (98.1)
mRS4-6, *n* (%)	16(1.9)

IQR: Inter quartile range.

### Result 2 comparison of temporal muscle thickness and CT values between normal and CSDH groups

The comparison of TMT and CT values between the normal group and the CSDH group is presented in Table 2. Significant differences in TMT were observed between the two groups. The mean left TMT was significantly lower in the CSDH group (5.00 ± 1.84 mm) than in the normal group (5.60 ± 1.71 mm) (*p* < 0.001). Similarly, the mean right TMT was significantly lower in the CSDH group (5.24 ± 1.89 mm) than in the normal group (5.61 ± 1.77 mm) (*p* < 0.01). The overall mean TMT was likewise significantly lower in the CSDH group (5.12 ± 1.74 mm) than in the normal group (5.60 ± 1.72 mm) (*p* < 0.001). However, CT values did not differ significantly between the two groups. The left, right, and mean CT values for the normal group were 47.21, 46.45, and 46.83, respectively, and for the CSDH group, they were 47.15, 46.89, and 47.02, respectively. The *p*-values for the CT values were 0.858, 0.768, and 0.901, indicating no significant differences between the normal and CSDH groups in terms of CT values. These findings suggest that TMT, rather than CT values, may be more closely associated with the development or progression of CSDH.

**Table 2:** Significant differences in temporal muscle thickness between normal group and CSDH group.

Parameters (*n* = 844)		TMT	*p*
	Normal (mean ± SD)	CSDH (mean ± SD)	
TMT			
Left	5.60 ± 1.71	5.00 ± 1.84	<0.001
Right	5.61 ± 1.77	5.24 ± 1.89	<0.001
Mean	5.60 ± 1.72	5.12 ± 1.74	<0.001
CT values			
Left	47.21 ± 19.47	47.15 ± 12.41	0.858
Right	46.45 ± 9.69	46.89 ± 12.45	0.768
Mean	46.83 ± 12.33	47.02 ± 11.71	0.901

TMT: temporal muscle thickness. *p*: *p* value of the Mann–Whitney test. SD: Standard Deviations.

### Result 3 characteristics and the side of CSDH occurrence according to temporal muscle thickness

In our multivariate analysis, patients with CSDH were categorized into three age groups (<41 years, 41–79 years, and >79 years), based on previous studies (9). Average TMT was a significant predictor of age classification (*p* < 0.001), whereas TMT difference showed no significant effect (*p* > 0.05). Univariate analysis revealed a significant association between TMT and gender. A greater TMT difference was positively associated with female sex (OR = 1.261, 95% CI: 1.048–1.411, *p* = 0.010), whereas males tended to have a higher average TMT (OR = 0.651, 95% CI: 0.574–0.737, *p* < 0.001). Additionally, TMT difference was strongly associated with the side of CSDH occurrence (*p* < 0.001), with CSDH more likely to occur on the side with lower TMT (OR = 0.219, 95% CI: 0.179–0.269). In contrast, average TMT did not significantly influence the side of CSDH occurrence (*p* = 0.411).

These findings suggest that across all age groups and genders, CSDH occurrence is significantly associated with the side of the thinner temporal muscle.

### Result 4 preexisting conditions and temporal muscle thickness in patients with CSDH

In our analysis, alcohol consumption, cardiac disease, and brain infarction were significantly associated with average TMT (*p* = 0.009, *p* = 0.006, and *p* < 0.001, respectively). Higher average TMT was associated with an increased likelihood of alcohol consumption (OR = 0.876, 95% CI: 0.793–0.967, *p* = 0.009). Similarly, greater average TMT was significantly associated with a lower likelihood of cardiac disease (OR = 1.442, 95% CI: 1.112–1.870, *p* = 0.006) and brain infarction (OR = 1.303, 95% CI: 1.141–1.487, *p* < 0.001). However, TMT difference and average TMT did not show significant associations with other preexisting conditions (Table 3).

**Table 3:** Uni- and multivariate analysis of juxtaposed characteristics according to TMT in CSDH.

Patient characteristics (*n* = 844)	TMT difference			Mean TMT		
	OR	95%CI	*p*	OR	95%CI	*p*
Age						
<40	-	-	-	-	-	-
40–79	1.118	0.888–1.407	0.343	2.400	1.968–2.926	<0.001
>79	0.967	0.816–1.145	0.694	1.739	1.483–2.026	<0.001
Sex	1.216	1.048–1.411	0.010	0.651	0.574–0.737	<0.001
Side of CSDH	0.219	0.179–0.269	<0.001	1.043	0.944–1.152	0.411
Preexisting conditions						
Trauma	0.972	0.874–1.080	0.599	1.018	0.938–1.104	0.670
Smoking	1.065	0.950–1.195	0.282	0.104	0.851–1.015	0.104
Drinking	1.014	0.891–1.154	0.834	0.876	0.793–0.967	0.009
Hypertension	1.011	0.910–1.123	0.844	0.977	0.901–1.059	0.570
Diabetes	1.119	0.988–1.268	0.077	1.033	0.938–1.138	0.512
Cardiac disease	1.162	0.853–1.581	0.341	1.442	1.112–1.870	0.006
Brain infarction	1.083	0.921–1.274	0.336	1.303	1.141–1.487	<0.001
Anticoagulation	0.867	0.741–1.015	0.077	0.899	0.794–1.018	0.094
Symptoms						
Headache	1.042	0.937–1.157	0.448	0.748	0.686–0.815	<0.001
Dizziness	0.959	0.856–1.075	0.472	1.048	0.959–1.144	0.299
Limb weakness	1.065	0.961–1.181	0.232	1.258	1.159–1.365	<0.001
Dysphasia	0.826	0.701–0.972	0.022	1.187	1.045–1.349	0.008
Disturbance of consciousness	1.170	0.915	0.211	1.143	0.939–1.391	0.183

OR: odds ratio; TMT Difference: (right-side TMT)–(left-side TMT).

### Result 5 symptoms at admission and temporal muscle thickness in patients with CSDH

In the univariate analysis, average temporal muscle thickness (TMT) was significantly associated with the presence of headaches, with lower average TMT linked to a higher likelihood of experiencing headaches (OR = 0.748, 95% CI: 0.686–0.815, *p* < 0.001). Similarly, average TMT was significantly associated with limb weakness; higher average TMT correlated with a lower likelihood of limb weakness (OR = 1.258, 95% CI: 1.159–1.365, *p* < 0.001). TMT difference was significantly associated with a higher likelihood of speech disorder (OR = 0.826, 95% CI: 0.701–0.972, *p* = 0.022), whereas higher average TMT was associated with a lower likelihood of speech disorder (OR = 1.187, 95% CI: 1.045–1.349, *p* = 0.008). However, neither TMT difference nor average TMT showed significant associations with other symptoms at admission (Table 3).

### Result 6 clinical outcome and temporal muscle thickness in patients with CSDH

Lower average TMT was significantly associated with a reduced risk of postoperative disturbance of consciousness (OR = 3.346, 95% CI: 1.129–9.918, *p* = 0.029). The mRS scores were dichotomized as follows: 0–3 indicating good functional outcomes and 4–6 indicating poor outcomes (severe disability or death). A lower average TMT was significantly associated with a higher likelihood of poor functional outcomes (mRS 4–6) (OR = 0.560, 95% CI: 0.391–0.804, *p* = 0.002). However, neither TMT difference nor average TMT showed significant associations with other postoperative complications or clinical outcomes (Table 4).

**Table 4:** Univariate analysis of clinical outcome according to TMT in CSDH.

Patient Characteristics (*n* = 844)	TMT difference			Mean TMT		
	OR	95%CI	*p*	OR	95%CI	*p*
Postoperative complication						
Postoperative seizures	1.089	0.650–1.823	0.747	1.361	0.890–2.082	0.155
Fever	0.867	0.618–1.216	0.407	1.198	0.918–1.563	0.184
Infection	1.270	0.551–2.923	0.575	1.050	0.541–2.039	0.844
Disturbance of consciousness	0.549	0.164–1.833	0.330	3.346	1.129–9.918	0.029
mRS	0.911	0.602–1.377	0.657	0.560	0.391–0.804	0.002
Recurrence	1.135	0.945–1.365	0.175	0.940	0.817–1.082	0.391

OR: odds ratio; TMT difference: (right-side TMT)–(left-side TMT).

## Discussion

The predictive value of TMT in patients with malignant brain tumors has been promisingly described in the literature, and previous studies have also highlighted its potential prognostic value in CSDH patients (27), which prompted the current investigation of patients from our institutional database. This study aimed to investigate the relationship between temporal muscle thickness on the side of chronic subdural hematoma occurrence and various clinical outcomes, including disease duration, prognosis, and recurrence. Our findings reveal several important insights and correlations.

Sarcopenia, a pathological condition characterized by low muscle strength, quantity, and quality, is closely associated with cerebrovascular diseases. Jauffret et al. found that presarcopenia and sarcopenia are independent risk factors for incident major adverse cardiac and cerebrovascular events (MACCEs) in a middle-aged and older Caucasian population (28). Sarcopenia is also associated with adverse cardiovascular outcomes, including increased mortality and morbidity (29, 30). Increasing attention has been directed toward the prognostic and predictive role of TMT in patients with various neurological conditions (31). In a study by Peball et al. (32), TMT seems to be a promising surrogate marker for sarcopenia and muscle strength in PD patients. Nagano et al. (33) also validated TMT measurement as an effective method for predicting sarcopenia in older patients after acute stroke. Similarly, Katsuki et al. (34) confirmed TMT as a prognostic factor in their multivariate analysis of older patients with SAH.

In our study, we first identified a significant association between TMT and both the occurrence and progression of CSDH. Further analysis confirmed that CSDH is more likely to develop on the side with thinner temporal muscles, suggesting that TMT could serve as a potential indicator for CSDH. Additionally, the significant inverse association between TMT and functional outcome, as assessed by the mRS, indicates that patients with thinner temporal muscles tend to have poorer prognosis. This finding is consistent with previous studies highlighting the influence of comorbidities and initial treatment efficacy on disease course and recurrence rates. Moreover, our analysis of factors associated with TMT revealed that age and certain preexisting conditions—notably cardiac disease and brain infarction—were significantly correlated with TMT. Older age and cardiac disease were associated with thinner temporal muscles, whereas a history of brain infarction was associated with greater TMT. These findings emphasize the multifactorial nature of muscle atrophy and its interaction with overall health, underscoring the need for a comprehensive approach to prognostic assessment in CSDH patients. In summary, our findings suggest that TMT is a significant prognostic indicator in CSDH, particularly for predicting functional outcomes.

Our analysis also found that neither TMT difference nor average TMT showed significant associations with other postoperative complications, such as recurrence or other clinical outcomes, beyond functional outcomes and postoperative consciousness disorder. This may be attributed to the multifactorial nature of CSDH, in which factors such as initial hematoma volume, patient age, and preexisting comorbidities likely play a more dominant role in determining long-term complications. In contrast, Charehsaz et al. examined the Relative Cortical Atrophy (RCA) Index as a predictor of recurrence after surgical evacuation of chronic subdural hematoma (CSDH). Their findings indicate that the RCA Index significantly predicts the risk of postoperative recurrence, with a higher degree of cortical atrophy associated with an increased likelihood of recurrence. Their study highlights the RCA Index as a precise predictor of CSDH recurrence, providing a well-defined cutoff value with high sensitivity and specificity (35). In comparison, our study provided a comprehensive analysis that incorporated sarcopenia and overall health status, thereby offering a more holistic perspective on patient prognosis. Whereas the RCA Index provides specificity in predicting recurrence, the assessment of TMT offers valuable insights into the broader health context and functional recovery, thereby serving as a complementary tool in the comprehensive management of patient outcomes. Future research involving larger, multi-center cohorts and longitudinal follow-up is warranted to validate these findings and elucidate the underlying mechanisms. Understanding the role of TMT in CSDH can enhance patient stratification and guide therapeutic decisions, ultimately improving clinical outcomes.

Our results demonstrated a significant inverse association between temporal muscle asymmetry and the side of hematoma occurrence, indicating that CSDH more frequently develops on the side with reduced temporal muscle thickness. This observation may be explained by a potential interaction between inflammatory processes and the temporalis muscle. Several studies have highlighted the role of inflammation in the formation and persistence of CSDH. Following minor trauma or cellular injury, inflammatory cells—including neutrophils, lymphocytes, macrophages, and eosinophils—are recruited to the subdural space. This recruitment promotes membrane formation and the development of fragile, permeable neovessels. Elevated levels of pro-inflammatory cytokines like IL-6 and IL-8 in CSDH fluid are associated with increased recurrence risk, while anti-inflammatory cytokines like IL-10 are linked to lower recurrence risk Elevated levels of pro-inflammatory cytokines such as IL-6 and IL-8 in CSDH fluid are associated with an increased recurrence risk, whereas anti-inflammatory cytokines like IL-10 are correlated with a lower recurrence risk (36–38). In parallel, inflammation is a known regulator of muscle protein metabolism. Pro-inflammatory cytokines, including C-reactive protein (CRP), interleukin-1 (IL-1), interleukin-6 (IL-6), and tumor necrosis factor-alpha (TNF-*α*), are key mediators of muscle degradation. They induce skeletal muscle mitochondrial dysfunction, leading to increased production of reactive oxygen species (ROS). The resulting oxidative stress, in turn, activates the ubiquitin-proteasome system, thereby accelerating muscle protein breakdown. Additionally, cytokines such as IL-6 can induce insulin resistance, which hinders the activation of the anabolic Akt/mTOR pathway and consequently impairs muscle protein synthesis (29, 30). However, whether the chronic inflammatory state associated with CSDH directly contributes to sarcopenia and a subsequent reduction in temporalis muscle thickness remains speculative and requires further investigation.

This study has several limitations that should be considered. First, although the study included a relatively large overall sample size, certain subgroups (e.g., specific age ranges or genders) had limited sample sizes, which may affect the generalizability and reliability of the findings for these populations. Second, the retrospective design is susceptible to selection and information biases. The findings are dependent on the quality and completeness of retrospectively collected data, and residual confounding due to unmeasured or imprecisely measured variables cannot be excluded. Third, it is important to note that TMT is a non-modifiable anatomical parameter, and its measurement can be influenced by numerous factors. The observed TMT asymmetry, while statistically significant, may be minimal in absolute magnitude and could be influenced by confounding variables such as age-related physiological changes, habitual chewing patterns (e.g., predominantly unilateral mastication), smoking history, nutritional status, and overall somatic sarcopenia. Although we adjusted for several variables in our analysis, residual confounding from unmeasured or imprecisely measured factors cannot be entirely ruled out. Therefore, TMT should not be interpreted as a standalone predictor but rather as a composite marker reflecting general health and frailty, rather than a direct causative factor in CSDH pathogenesis. Fourth, the study primarily assessed short-term postoperative outcomes and lacked long-term follow-up data, which limits insights into the role of TMT in long-term prognosis. Fifth, our cohort primarily consisted of patients with a history of head trauma, which aligns with the most common etiology of CSDH. Cases of non-traumatic CSDH, such as those secondary to spontaneous intracranial hypotension, high-altitude exposure, or other rare causes (39), were not specifically identified or analyzed. Consequently, the applicability of our findings to non-traumatic CSDH etiologies remains uncertain and warrants investigation in future studies. Sixth, although the study explored the role of inflammation in CSDH, the complexity and multifactorial nature of the associated inflammatory processes may not have been fully captured. Thus, further molecular and mechanistic studies are required to elucidate the underlying pathways. Finally, the single-center design may introduce regional and practice pattern biases, thereby limiting the generalizability of the results to other healthcare settings. These limitations highlight the need for larger, multicenter, prospective studies with long-term follow-up to validate and extend our findings.

## Conclusion

This study identified TMT as a significant prognostic marker in patients with CSDH, wherein thinner muscles were associated with poorer clinical outcomes. Although TMT was not predictive of recurrence or other specific complications, it served as an effective indicator of functional prognosis. The complex role of inflammation in CSDH necessitates further research to validate these findings, elucidate the underlying mechanisms, and refine patient management strategies.

## Data Availability

The raw data supporting the conclusions of this article will be made available by the authors, without undue reservation.

## Footnotes

1
http://www.neusoft.com
